# Phytochemical Analysis and Anti-Inflammatory and Anti-Osteoarthritic Bioactive Potential of *Verbascum thapsus* L. (Scrophulariaceae) Leaf Extract Evaluated in Two In Vitro Models of Inflammation and Osteoarthritis

**DOI:** 10.3390/molecules26175392

**Published:** 2021-09-05

**Authors:** Giovanna Calabrese, Agata Zappalà, Anna Dolcimascolo, Rosaria Acquaviva, Rosalba Parenti, Giuseppe Antonio Malfa

**Affiliations:** 1 Department of Chemical, Biological, Pharmaceutical and Environmental Sciences, University of Messina, 98166 Messina, Italy; 2 Department of Biomedical and Biotechnological Sciences, Physiology Section, University of Catania, 95123 Catania, Italy; azappala@unict.it (A.Z.); anna.dol@alice.it (A.D.); parenti@unict.it (R.P.); 3 Department of Drug and Health Sciences, Section of Biochemistry, University of Catania, 95125 Catania, Italy; racquavi@unict.it

**Keywords:** verbascoside, phenylethanoid glycosides, phenylpropanoids, inflammatory cytokines, human chondrocytes, cartilage, matrix metalloproteases, iNOS, COX-2

## Abstract

Osteoarthritis (OA) is a complex disease, source of pain and disability that affects millions of people worldwide. OA etiology is complex, multifactorial and joint-specific, with genetic, biological and biomechanical components. Recently, several studies have suggested a potential adjuvant role for natural extracts on OA progression, in terms of moderating chondrocyte inflammation and following cartilage injury, thus resulting in an overall improvement of joint pain. In this study, we first analyzed the phenylethanoid glycosides profile and the total amount of polyphenols present in a leaf aqueous extract of *Verbascum thapsus* L. We then investigated the anti-inflammatory and anti-osteoarthritic bioactive potential of the extract in murine monocyte/macrophage-like cells (RAW 264.7) and in human chondrocyte cells (HC), by gene expression analysis of specifics inflammatory cytokines, pro-inflammatory enzymes and metalloproteases. Six phenylethanoid glycosides were identified and the total phenolic content was 124.0 ± 0.7 mg gallic acid equivalent (GAE)/g of extract. The biological investigation showed that the extract is able to significantly decrease most of the cellular inflammatory markers, compared to both control cells and cells treated with *Harpagophytum procumbens* (Burch.) DC. ex Meisn, used as a positive control. *Verbascum thapsus* leaf aqueous extract has the potential to moderate the inflammatory response, representing an innovative possible approach for the inflammatory joint disease treatment.

## 1. Introduction

Osteoarthritis (OA), also known as degenerative arthritis, is the most common chronic condition of joint disease that affects up to 15% of the adult population. It is a disorder caused in part by damage of cartilage component and function, and deregulation of pro-inflammatory and anti-inflammatory pathways [1,2,3]. OA is mainly characterized by the breakdown of the articular cartilage and often accompanied by subchondral bone injuries, deterioration of tendons and ligaments and several levels of inflammation of the synovium, leading to pain, swelling and disability. OA is an incurable disease because once cartilage breaks down, it can hardly be repaired, but some therapies can slow down their breakdown, alleviate pain and improve joint mobility [4]. In fact, pharmacological therapies, with anti-inflammatory and chondroprotective effects, aim at reducing pain and preserving matrix integrity, thus improving the patient’s joint activity and quality of life. Recently, several studies have suggested a potential adjuvant role for natural extracts, such as curcumin, resveratrol, pomegranate, as well as genistein and soy protein, on OA progression, in terms of moderating chondrocyte inflammation and following cartilage injury resulting in the improvement of joint pain [5,6]. Moreover, the traditional use of *Harpagophytum procumbens* (Burch.) DC. ex Meisn. (*H. procumbens*), belonging to the Pedaliaceae family, better known as devil’s claw, for the relief of articular pain is well documented and the analgesic and anti-inflammatory activities of different root extracts and the iridoid harpagoside have been extensively investigated in several experimental models [7,8,9,10]. *Verbascum thapsus* L. (*V. thapsus*), commonly known as common mullein, is a Eurasian plant belonging to the Scrophulariaceae family. It is a monocarpic and biennial herb that largely grows in cliffs, meadows, fields and ridges on dry, sandy and rocky soils. The plant forms a low vegetative rosette in the first year, characterized by several hairy elliptic-lanceolate cauline and alternate leaves. During the second growing season, from early spring to late summer, the plant forms a raceme inflorescence composed of small yellow sessile flowers densely grouped on an indeterminate spike up to 2 m tall. In Sicily, it can be easily found, on cultivated lands, along roads and in rocky areas over the altitude of 1000 m a.s.l. [11]. *V. thapsus* is widely used in traditional medicine as a medicinal herb since the year dot. Leaves and flowers extracts have been used as a domestic medication against inflammatory diseases, asthma, pulmonary disorders, migraine, fever, congestion, allergies and colic, including arthritis and osteoarthritis [12,13]. In fact, plants from the genus Verbascum are a source of a wide variety of chemical constituents, such as iridoids, saponins, flavonoids, phenolic acids and phenylethanoid glycosides (PhEGs), a class of water-soluble compounds of which the most known is verbascoside [14]. This compound was isolated for the first time in 1950 from *Verbascum sinuatum* L., from which it got its name, even if it was already known with the name of acteoside [15]. Verbascoside has extensively been characterized as an effective scavenger of biologically active free radicals and an anti-inflammatory and wound healing agent both in vitro and in vivo [16]. Recently, interest in PhEGs has been growing and several studies described various beneficial biological effects involved in the prevention and treatment of various human diseases [17]. The present study is aimed at investigating the bioactive potential of an aqueous leaf extract of *V. thapsus*, firstly by determining the Total Phenolic Content and the PhEGs profile and, subsequently, evaluating the possible anti-inflammatory and anti-osteoarthritic activities compared to an *H. procumbens* standardized extract used as a positive control, in both inflammatory (LPS-induced leukemic monocyte-macrophage) and osteoarthritic (IL-1β-stimulated human adult chondrocytes) in vitro model. The gene expression analysis of specifics inflammatory cytokines (Interleukin (IL)-1β and 6, tumor necrosis factor-alpha (TNF-α)), pro-inflammatory enzymes (cyclooxygenase-2 (COX-2), inducible nitric oxide synthase (iNOS)) and matrix-metalloproteinase (MMP)-1, -8, -9 and -13 was performed.

## 2. Results

### 2.1. Phenylethanoid Glycosides Profile and Total Phenolic Content

**Figure 1:**
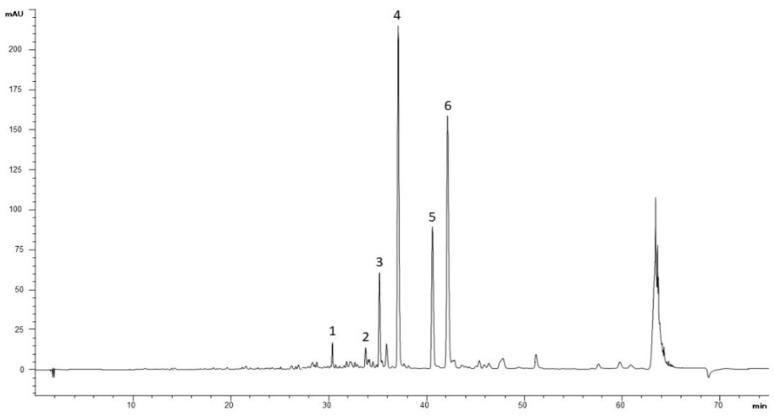
HPLC-DAD phenylethanoid glycosides fingerprint of *V. thapsus* leaf aqueous extract. Column: Ascentis Express C18, 15 cm × 4.6 mm, 2.7 µm d.p. The numbers indicating peaks refer to the identified and quantified compounds reported in Table 1.

The trace recorded at 330 nm (Figure 1 and Figure S1) showed four predominant constituents (peaks 3, 4, 5 and 6), accompanied with at least another two minor peaks (peaks 1 and 2), six compounds were identified. Peak 1 was identified as samioside (52.3 mg/mL), peak 2 was identified as echinacoside (42.1 mg/mL), peak 3 was identified as forsythoside B (108.1 mg/mL), peak 4 was identified as verbascoside (254.8 mg/mL), peak 5 was identified as iso-verbascoside (142.9 mg/mL) and peak 6 was identified as martynoside (118.4 mg/mL) (Table 1). The total PhEGs content determined by HPLC was 718.6 (mg/mL) calculated using calibration curves with the closest appropriate standard. Otherwise, all the other peaks not identified are mostly attributable to different classes of polyphenols. Therefore, the total phenolic content determined spectrophotometrically by the Folin–Ciocalteu method, was estimated in 124.0 ± 0.7 mg gallic acid equivalent (GAE)/g of extract.

### 2.2. Evaluation of the Cytotoxic Effect of V. thapsus Extract on RAW 264.7 Cell Line

**Figure 2:**
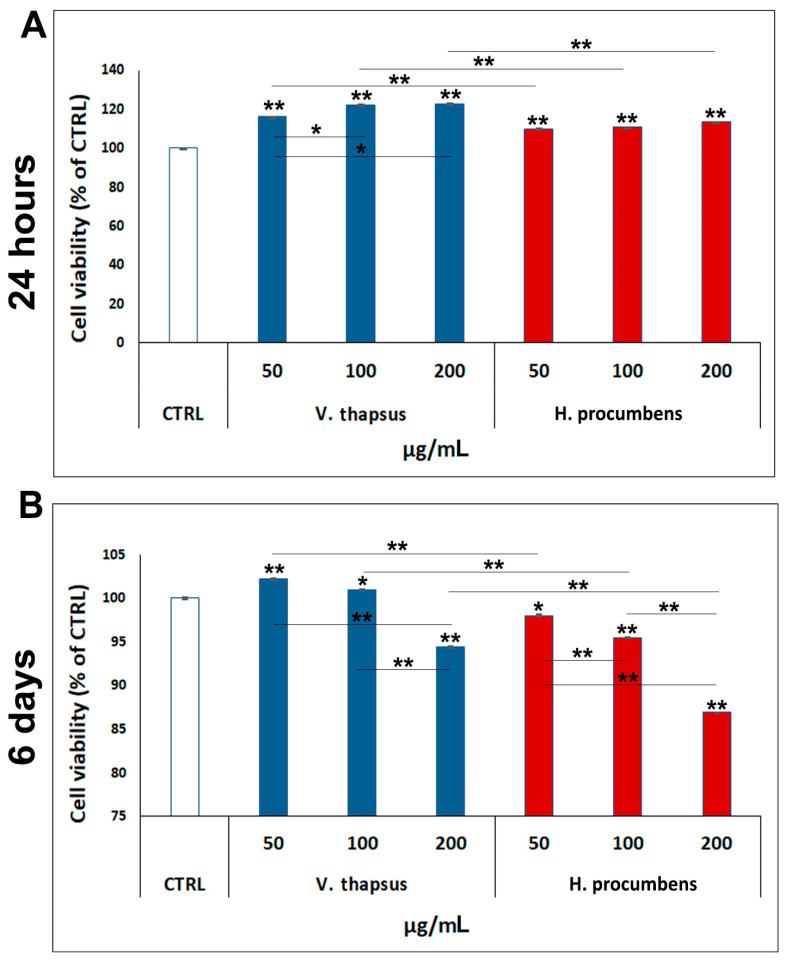
Cytotoxic effect of *V. thapsus* on RAW 264.7 cells. MTT test performed on RAW 264.7 cells treated with *V. thapsus* and *H. procumbens* extracts (50, 100 and 200 µg/mL) for 24 h and 6 days. *H. procumbens* = positive control. Data are represented as the means ± SD of three independent experiments. ANOVA test *p*-values were reported and (** *p* < 0.01 and * *p* < 0.05) indicate significant differences between the two groups, as reported by the Holm post-hoc test.

In order to determinate the most appropriate concentration of *V. thapsus* to use in our in vitro study, we firstly evaluated the cytotoxic effects of three different concentrations of extract (50, 100 and 200 µg/mL) on RAW 264.7 cell line compared to *H. procumbens*, used as positive control. After, a cell viability assay was performed at two several time points from treatment (24 h and 6 days) (Figure 2A,B). The data reported in Figure 2A show that neither *V. thapsus* and *H. procumbens* were cytotoxic for RAW 264.7 after 24 h of treatment, at all the concentrations used, but on the contrary, treatment with both extracts seems to stimulate proliferation. Specifically, cell viability values were: 50 µg/mL = (116 ± 0.02)%, 100 µg/mL = (122 ± 0.09)%, 200 µg/mL = (123 ± 0.02)% for *V. thapsus*; 50 µg/mL = (110 ± 0.01)%, 100 µg/mL = (111 ± 0.01)%, 200 µg/mL = (114 ± 0.02)% for *H. procumbens*, compared to 100% of the control. Differently, after 6 days of treatment, while the *V. thapsus* showed a weak cytotoxic effect (about 5.8 ± 0.03%) only at the highest concentration (200 µg/mL), *H. procumbens* exhibits increasing cytotoxicity from the concentration of 50 µg/mL to 200 µg/mL ((1.1 ± 0.01)% < (4.7 ± 0.006)% < (13,3 ± 0.07)%). More in detail, cell viability percentages, compared to 100% of the control, were: 50 µg/mL = (102 ± 0.10)%, 100 µg/mL = (101 ± 0.02)%, 200 µg/mL = (95 ± 0.05)% for *V. thapsus*; 50 µg/mL = (98 ± 0.11)%, 100 µg/mL = (95 ± 0.03)%, 200 µg/mL = (87 ± 0.02)% for *H. procumbens*. Since both *V. thapsus* and *H. procumbens* showed a greater cytotoxic effect at the concentration of 200 µg/mL, we decided to use only the 50 and 100 µg/mL concentrations, for the following experiments.

### 2.3. Evaluation of the Anti-Inflammatory Property of V. thapsus Extract on RAW 264.7 Cells

**Figure 3:**
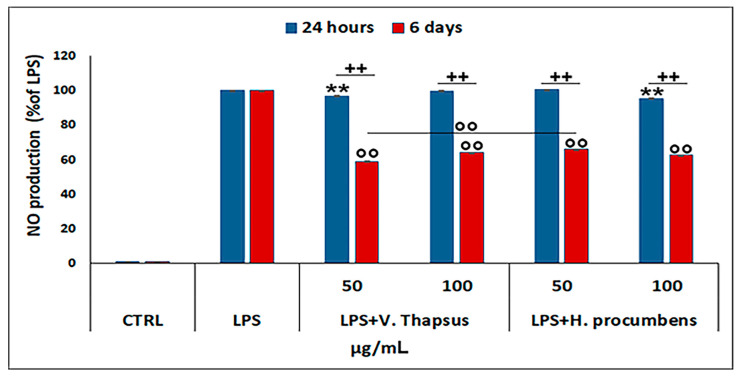
Effect of *V. thapsus* on nitric oxide production in RAW 264.7 cells. Griess assay performed on the supernatant of LPS-stimulated RAW 264.7 treated with *V. thapsus* and *H. procumbens* extracts (50 and 100 µg/mL) for 24 h and 6 days. *H. procumbens* = positive control. Data are represented as the means ± SD of three independent experiments. ANOVA test *p*-values were reported (*p* < 0.0001), and (**, ^++,^ °° *p* < 0.01) indicate significant differences between the two groups, as reported by the Holm post hoc test.

To assess the anti-inflammatory property of *V. thapsus* compared to *H. procumbens* the two selected concentrations of extract (50 and 100 µg/mL) were added on LPS-stimulated RAW 264.7 and NO• levels measured after 24 h and 6 days of treatment. Our data showed that after 24 h of treatment, only *V. thapsus* at the concentration of 50 µg/mL and *H. procumbens* at the concentration of 100 µg/mL were able to reduce the NO• level in a statistically significant manner. On the contrary, after 6 days of treatment, *V. thapsus* and *H. procumbens*, at both concentrations, were able to significantly decrease the NO• level compared to the control (** *p* < 0.01), even if *V. thapsus* at the concentration of 50 µg/mL showed a greater NO• level reduction than *H. procumbens* at the same concentration of 50 µg/mL (** *p* < 0.01) (Figure 3).

### 2.4. Evaluation of the Cytotoxic Effect of V. thapsus Extract on HC Cell Line

**Figure 4:**
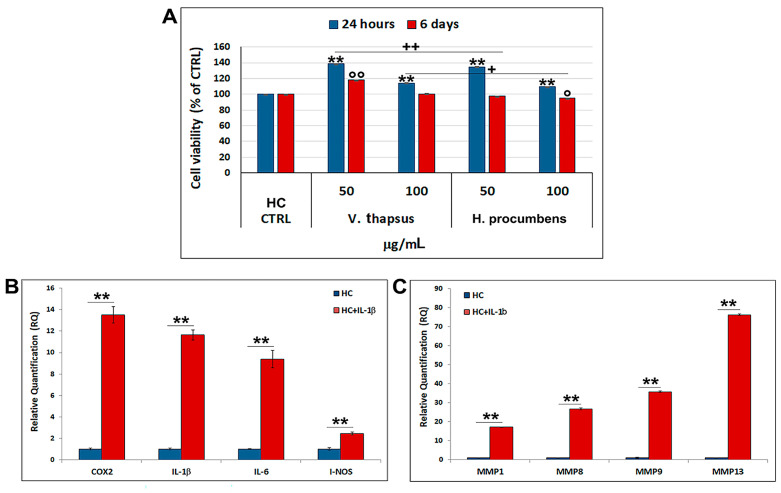
(**A**) Cell viability test performed on HC cells treated with *V. thapsus* and *H. procumbens* extracts (50 and 100 µg/mL) for 24 h and 6 days. *H. procumbens* = positive control. (**B**,**C**) Gene expression profile of IL-1β-stimulated HC with specifics inflammatory mediators and metalloproteases. Data are represented as the means ± SD of three independent experiments. ANOVA test *p*-values were reported (*p* < 0.0001) and (**, ^++^, °° *p* < 0.01 and ^+^, ° *p* < 0.05) indicate significant differences between the two groups, as reported by the Holm post hoc test.

Before evaluating the anti-inflammatory effect of *V. thapsus* on the cartilage in vitro, we performed a cytotoxicity study, also on the HC cells at the selected concentrations (50 and 100 µg/mL). The effects of *V. thapsus* on the HC cell viability are shown in Figure 4A. Our data reported that *V. thapsus*, both at the two concentrations used and at the times analyzed, does not affect cell viability, but rather significantly improves cell growth compared to both control and *H. procumbens* (** *p* < 0.01 and * *p* < 0.05). Specifically, the percentage values of cell viability, compared to 100% of the control, after 24 h and 6 days of treatment, were: 50 µg/mL = (138 ± 0.05)% and 100 µg/mL = (114 ± 0.05)%, 50 µg/mL = (118 ± 0.12)% and 100 µg/mL = (101 ± 0.05)% for *V. thapsus*, respectively; 50 µg/mL = (135 ± 0.13)% and 100 µg/mL = (110 ± 0.05)%, 50 µg/mL = (97 ± 0.08)% and 100 µg/mL = (95 ± 0.05)% for *H. procumbens*, respectively.

To assess whether IL-1β was able to induce activation of the inflammatory response in chondrocytes stimulated for 24 h, we performed a gene expression profile by using specific inflammatory mediators and metalloproteases. The results, reported in Figure 4B,C, highlight that IL-1β-stimulated HC show a significant expression level increase of all analyzed genes compared to not stimulated cells. Precisely, IL-1β-stimulated HC show an increase of 13.5-fold for COX2, 11.6-fold for IL-1β, 9.4-fold for IL-6, 2.5-fold for I-NOS, 17.1-fold for MMP1, 26.8-fold for MMP3, 34.5-fold for MMP9 and 75.6-fold for MMP13.

### 2.5. Anti-Osteoarthritis Effect of V. thapsus Extract

**Figure 5:**
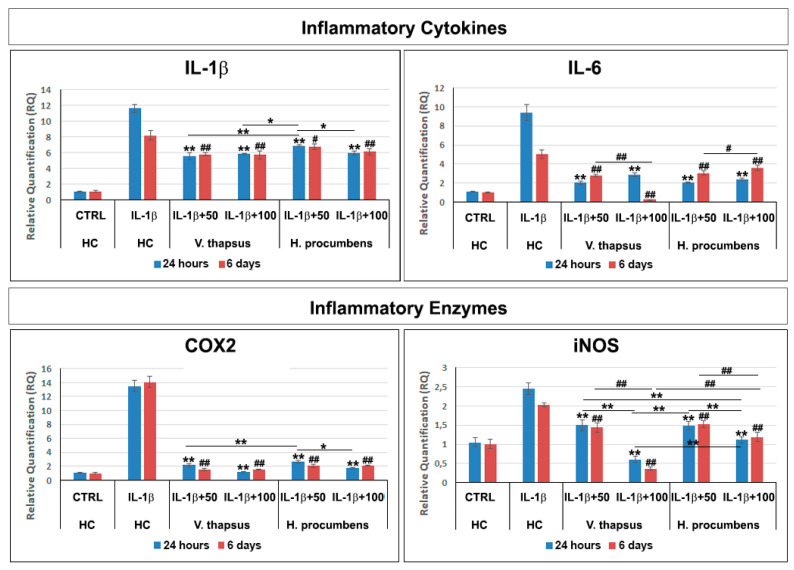
Effects of *V. thapsus* and *H. procumbens* treatment on IL-1β-induced expression levels of inflammatory cytokines and enzymes. Data are represented as the means ± SD of three independent experiments. ANOVA test *p*-values were reported (*p* < 0.0001) and (**, ^##^ *p* < 0.01 and *, ^#^ *p* < 0.05) indicate significant differences between the two groups, as reported by the Holm post hoc test.

**Figure 6:**
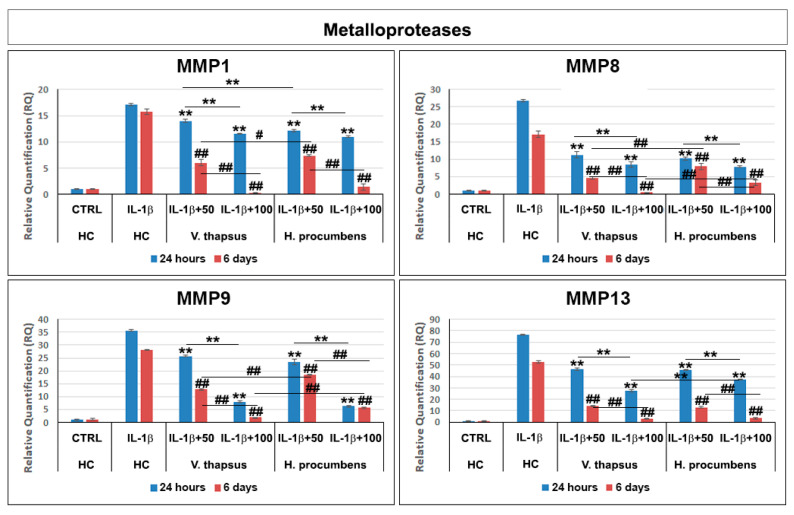
Effects of *V. thapsus* and *H. procumbens* treatment on IL-1β-induced expression levels of inflammatory cytokines and enzymes. Data are represented as the means ± SD of three independent experiments. ANOVA test *p*-values were reported (*p* < 0.0001) and (**, ^##^ *p* < 0.01 and ^#^ *p* < 0.05) indicate significant differences between the two groups, as reported by the Holm post hoc test.

To evaluate the anti-osteoarthritis effect of *V. thapsus* on IL-1β-stimulated HC, we performed a gene expression profile with specifics inflammatory mediators (Figure 5) and MMPs (Figure 6) after 24 h and 6 days of treatment. Our results showed that after 24 h and 6 days of *V. thapsus* treatment, at both concentrations (50 and 100 µg/mL), it was possible to observe a significant decrease (* *p* < 0.05 and ** *p* < 0.01) in the expression levels of all cytokines, pro-inflammatory enzymes and metalloproteases, even if the higher concentration displayed a greater effect (Figure 5) compared to both the control (IL-1β-stimulated HC) and HC cells treated with *H. procumbens.*

## 3. Discussion

Osteoarthritis (OA) is a complex disease, source of pain and disability that affects millions of people worldwide. There is no single cause for OA, and the exact etiology is complex, multifactorial and joint-specific, with genetic, biological and biomechanical components [18]. Recent in vitro and preclinical studies suggest the protective effects of polyphenols and a possible key role in the prevention and treatment of the early stages of OA, through the mitigation of chondrocyte inflammation and joint-associated tissues [6,19,20]. In vitro and clinical studies showed that both *H. procumbens* and its bioactive secondary metabolites, the iridoid harpagoside, exert some anti-inflammatory effects and can improve pain and movement limitation in subjects with OA in the lower extremities [7,8]. Several evidences demonstrated that phenylethanoids can modulate different molecular pathways underlying inflammatory responses in human cells in vitro and inhibit inflammation in different tissues in vivo [21,22]. Based on this evidence, in this study, we demonstrated the inhibitory effects against inflammation of a leaf extract of *V. thapsus*, compared to a standardized extract of *H. procumbens* (harpagoside 12%), on the major inflammation signaling pathways in LPS-activated RAW 264.7 and in IL-1β activated HC cells [23]. After characterizing the profile and amount of PhEGs and the total polyphenol content of the extract, which were estimated in 9.92% (W/W) and 124.0 ± 0.7 mg gallic acid equivalent (GAE)/g of extract, respectively, we found that both extracts were not toxic at all concentrations tested after 24 h, but rather, they seemed to stimulate cell proliferation. Conversely, after 6 days of treatment, *H. procumbens* exerted a dose dependent cytotoxicity, while *V. thapsus* showed only a slight cytotoxic effect (5.8 ± 0.03) % at the higher dosage of 200 µg/mL in RAW 264.7 cells (Figure 2A,B). Macrophages play an important role in the initiation, maintenance and resolution of inflammation. In fact, during inflammation, infiltrating macrophages and neutrophils release nitric oxide (NO•), which is known to mediate the inflammatory response in tissues [24,25]. Inflammatory responses in macrophages induced by LPS also include increased expression of iNOS with consequent production of NO• [26]. Subsequently, we first focused on the LPS-induced NO• production, demonstrating that both extracts were able to significantly decrease NO• production after 6 days of pre-treatment (about 40%), while after 24 h, no significant change in NO• levels were detected in RAW 264.7 cells (Figure 3). Therefore, *V. thapsus* anti-inflammatory properties were similar to those of *H. procumbens*, but without concomitant cytotoxic effects, according to previous results about *V. thapsus* extracts or PhEGs compounds, performed in different experimental models [12,14,17,27,28]. After confirming the anti-inflammatory effect of the extract on macrophages, we proceeded to assess the inhibitory action on the cartilage inflammatory process in an in vitro model. In osteoarthritic condition, chondrocytes express IL-1, which induces the expression of MMPs and TNF-α. IL-1 and TNF-α increase the prostaglandin E_2_ (PGE_2_) synthesis by stimulating the COX-2 gene expression and upregulate the production of nitric oxide via iNOS [2,18,19]. In addition, IL-1β and TNF-α can also induce other proinflammatory cytokines, including IL-6 [29]. Based on this evidence, we evaluated the expression levels of cytokines and pro-inflammatory enzymes and metalloproteases involved in the inflammatory process of OA. Our qRT-PCR data showed that *V. thapsus* and *H. procumbens* extracts, at the used concentrations (50 and 100 µg/mL) and at both time points (24 h and 6 days), are able to decrease the expression levels of IL-1β and IL-6 pro-inflammatory cytokines, together with the inflammatory enzymes COX-2 and iNOS, compared to IL-1β-induced HC. However, *V. thapsus* extract exhibited a greater anti-inflammatory effect on IL-6, COX-2 and iNOS at the concentration of 100 µg/mL for 6 days, compared to *H. procumbens* extract at all concentrations and time points analyzed (Figure 5). The present study’s obtained data agreed with the several pieces of evidence reported in the scientific literature, showing that PhEGs can differently modulate MAPK, NF-κB, JAK-STATs and Nrf2 pathways, providing inhibitory effects on different inflammatory mediators, such as TNF-α, IL-6, NO and ROS generation [28]. Furthermore, it was observed that the presence of two adjacent hydroxide groups in PhEGs molecular structures, as it is for most PhEGs compounds present in our extract (Table S1), is probably linked to a more marked anti-inflammatory activity [30]. Matrix metalloproteinases (MMPs) are well established to play key roles in OA through degradation of the extracellular matrix, inducing degenerative changes in joint cartilage [31,32]. In our in vitro experimental model of OA, both extracts decreased mRNA expression levels of the examined MMPs induced by the IL-1β exposure, but in particular, the PhEGs compounds present in the extract at the concentration of 100 µg/mL for 6 days, markedly reduced mRNA levels of MMP1, MMP8 and MMP9 compared to *H. procumbens* extract at the same experimental condition (Figure 6). These preliminary results, although they require further deep studies, suggest for the first time that *V. thapsus* leaf extract phytocomplex, with its anti-inflammatory activity, has the potential to counteract the response of HC to the inflammatory insults and tissue degeneration that typically characterize the osteoarthritic disease.

## 4. Materials and Methods

### 4.1. Chemicals and Drugs

All solvents and chemicals were purchased from Sigma-Aldrich (Milano, Italy) except when otherwise specified. The *Harpagophytum procumbens* (Burch.) DC. ex Meisn. dry extract was kindly provided by Bionap (Belpasso, Catania, Italy), it was a commercial sample standardized to the content of harpagoside (12%) determined by HPLC analysis. The reference compounds used in the *V. thapsus* HPLC analysis were samioside (internally purified to purity of 80% by HPLC), forsythoside B (purity of 95% by HPLC), verbascoside (purity of 91% by HPLC), isoverbascoside (purity of 84% by HPLC), martynoside (purity of 98% by HPLC) (PhytoLab GmbH & Co. Vestenbergsgreuth, Germany) and echinacoside (purity 98% by HPLC) (Sigma-Aldrich, Milano, Italy).

### 4.2. Plant Material and Extraction Procedure

The final extract called Verbalief^®^ (product batch number EXPS-01201907-115), was performed and provided by Bionap (Belpasso, Catania, Italy). *Verbascum thapsus* L. leaves (medicinal name has been unified using the Kew Medicinal Plant Names Services, https://www.kew.org, accessed on 23 August 2021) were collected at the beginning of the second year of growing before the beginning of the flowering stage, in a cultivated field in the area of Mount Etna (37°35′34″ N, 14°58′27″ E, Catania, Italy). The taxonomic identification was confirmed by the pharmaceutical botanist Prof. R. Acquaviva, Department of Drug and Health Sciences, University of Catania (Italy). A voucher specimen (No. 02/19) was deposited in the same department. After harvesting, the plant material was cleaned and dried at 40 °C for 72 h in a ventilated oven. Subsequently, it was ground and blended before the extraction that was performed in ten times the volume of osmotized water at 80 °C under continuous stirring for five hours. The obtained solution was filtered and concentrated; the yield of the leaf extract, compared to 100 g of dried plant material, was about 30.00%. The extract was subsequently resuspended in water and supported on maltodextrin by spray drying.

### 4.3. Determination of Total Phenolic Content

The total phenolic content of *V. thapsus* leaf extract was determined spectrophotometrically by the Folin–Ciocalteu method, as previously described by Genovese [33], and compared to a calibration curve of a known amount of gallic acid used as standard. The absorbance was measured at 765 nm with a UV-1601 spectrophotometer (Shimadzu, Milan, Italy). The total polyphenols were estimated as gallic acid equivalent (GAE) and expressed in mg GAE/g extract (dw) ± standard deviation (SD). The data were obtained from three independent determinations.

### 4.4. Determination of Phenylethanoid Glycosides by HPLC-DAD

The polyphenolic fingerprinting of the extract was defined by high-pressure liquid chromatography (HPLC). The analyses were performed as above described [34]. Briefly, samples were dissolved in 1 mL of dimethylformamide/water solution (9:1) with a final concentration of 10 mg/mL. HPLC-DAD analyses were carried out in duplicate and performed using a Shimadzu LC 20, equipped with a diode array detector (DAD) and with a 150 × 4.6 mm i.d., 2.7 µm Ascentis Express C 18 column; the mobile phases: H_2_O/H_3_PO _4_(99:1, solvent A), MeOH/CAN/H3PO4 (49,5:49,5:1, solvent B); the gradient used was: concentration of the solvent A of 95% going to 77% (34 min), maintained at 77% (3 min), 74% (60 min), 60% (85 min), 20% (90 min) and 0% (92 min); total time 105 min. The column temperature was maintained at 25 °C. The flow was 1 mL/min and the injection volume was 5 μL. The chromatogram profiles were recorded from 190 to 500 nm and monitored at 280 and 330 nm ± 2 nm and the quantitative analysis was performed at wavelengths of 330 nm ± 2 nm.

### 4.5. Cell Cultures

Two different cell lines, mouse leukemic monocyte-macrophage (RAW 264.7, Sigma-Aldrich, Milan, Italy) and human adult chondrocytes (HC, Sigma-Aldrich) were used in this study. RAW 264.7 cells were cultured in EMEM (Sigma-Aldrich) supplemented with 2 mM L-Glutamine (Euroclone, Milan, Italy), 1% Non-Essential Amino Acids (Sigma-Aldrich), 10% Fetal Bovine Serum (FBS, Sigma-Aldrich) and penicillin/streptomycin/amphotericin (PSA) (Euroclone), while HC cells were grown in Chondrocyte Growth Medium (Sigma-Aldrich) containing PSA. Cells were maintained in a humidified environment at 37 °C and 5% CO_2_/95% air atmosphere and cultured in T75 flasks. The medium was replaced twice a week and cells were split at about 60–80% of confluence. Treatments with *V. thapsus* and *H. procumbens* were performed by adding different concentrations of each extract (50, 100 and 200 µg/mL) to the culture medium for 24 h and 6 days before inducing inflammation in cells with LPS (1 µg/mL) (O26:B6 E. coli, Sigma-Aldhric) and Il-1β (10 ng/mL) (recombinant human Il-1ß (PeproTech EC, London, UK)). Next, the medium was removed and further analyses were carried out.

### 4.6. Cell Viability Assays

The cytotoxic effect of the *V. thapsus* was evaluated by colorimetric assay MTT [3-(4,5-dimethylthiazol-2-yl)-2,5-diphenyltetrazolium bromide] (Sigma-Aldrich), as previously reported [35]. For MTT 5 × 10^3^ cells/well were cultured in 96 wells and after 24 h, a fresh medium containing several concentrations of *V. thapsus* and *H. procumbens* was added. Following 24 h and 6 days of incubation, the medium was removed, LPS (1 µg/mL) (O26:B6 E. coli, Sigma-Aldhric) and Il-1β (10 ng/mL) (recombinant human Il-1ß (PeproTech EC, London, UK)) was added for 24 h. After the cells were washed and 200 µL of MTT solution (1 mg/mL in FBS-free medium) was added to each well and incubated for 2 h at 37 °C and 5% CO_2_. Following 2 h of incubation, the medium was removed, each well was washed two times using cold PBS, and the formed crystals were melted using 200 μL of DMSO. The absorbance at 570 nm was read using a synergy HT plate reader (BioTek Instruments, Inc., Winooski, VT, United States).

### 4.7. NO• Production

The inhibitory effect of *V. thapsus* on NO• production was determined by measuring nitrite levels using the Griess reagent (Sigma-Aldrich) according to manufacturer instructions and as previously described [36,37]. Briefly, LPS-induced RAW 264.7 cells were cultured in a medium containing *V. thapsus* (50 and 100 µg/mL) for 24 h and 6 days. After 24 h and 6 days of treatment, 100 µL of the culture medium was mixed with an equal volume of Griess reagent and incubated at room temperature for 10 min according to manufacturer instructions. The nitrite content in culture media was determined at 540 nm using a synergy HT plate reader (BioTek Instruments, Inc.).

### 4.8. Quantitative Real-Time PCR (qRT-PCR)

For qRT-PCR analyses, total RNA, from LPS-induced RAW 264.7 and Il-1β-stimulated HC cells treated with *V. thapsus*, was isolated using Rneasy Mini Isolation Kit (Qiagen, Germantown, MD, USA) and quantified as previously described [38,39]. Three independently isolated and cultured samples were used. cDNA was synthesized from 1 μg of total RNA using ImProm-II Reverse Transcription System (Promega, Milan, Italy). Quantitative PCR was performed using SYBR Green method on a 7900HT Real Time PCR (Applied Biosystems). Specific primers for each of the investigated molecular endpoints were designed using primer blast and selecting exon-exon junctions on mRNA as a target region for annealing. Each sample was tested in triplicate and gene expression was assessed using the 2-ΔΔCt method. RNA from control cells was used as a reference for relative expression quantitation. The following PCR primers were used: IL-1β, IL-6, iNOS, COX2 and MMPs (1, 8, 9 and 13). Oligonucleotide sequences are reported in Table 2. Results were normalized to the levels of Glyceraldehyde 3-Phosphate Dehydrogenase (GAPDH).

### 4.9. Statistical Analysis

Statistical analyses were performed by one-way and two-way ANOVA. Holm methods have been used as post hoc tests when the ANOVA reported statistically significant differences, to evaluate the differences between the individual time-points or treatment groups. For all experiments, * *p* < 0.05 was considered to be significant.

## 5. Conclusions

In conclusion, most of the results obtained in this study, based on an in vitro model of OA, are comparable to those exerted by *H. procumbens* extract, but with fewer cytotoxic effects. In addition, for some of the considered biomarkers, such as IL-6, COX-2, iNOS and MMPs 1, 8 and 9, the inhibitory effects resulted more remarkably, demonstrating that *V. thapsus* leaf aqueous extract exhibits a good anti-inflammatory activity and suggesting that this extract, which certainly needs to be supported by further in-depth studies, could be a new potential candidate in the treatment of the early stages of osteoarthritis or mild joint inflammation.

## Figures and Tables

**Table 1:** Phenylethanoid glycosides identified and quantified in *V. thapsus* leaf aqueous by HPLC-DAD.

Peack	Compound	Wavelength (nm)	Ret. Time (min)	Content (mg/mL)
1	samioside	330	30.37	52.3
2	echinacoside	330	33.80	42.1
3	forsythoside B	330	35.23	108.1
4	verbascoside	330	37.12	254.8
5	iso-verbascoside	330	40.61	142.9
6	martynoside	330	42.10	118.4

**Table 2:** qRT-PCR primer sequences.

Target	Forward	Reverse
Il-1ß	GGAGAATGACCTGAGCACCT	GGAGGTGGAGAGCTTTCAGT
Il-6	AGTCCTGATCCAGTTCCTGC	CTACATTTGCCGAAGAGCCC
iNOS	ACAGCACATTCAGATCCCCA	GCCGAGATTTGAGCCTCATG
COX2	TAGTACTCCCGGTTGAAGCC	ACGATGGGCATGAAACTGTG
MMP1	CTGAAGGTGATGAAGCAGCC	AGTCCAAGAGAATGGCCGAG
MMP8	ATGGACCAACACCTCCGCAA	GTCAATTGCTTGGACGCTGC
MMP9	CGCAGACATCGTCATCCAGT	GGATTGGCCTTGGAAGATGA
MMP13	CTATGGTCCAGGAGATGAAG	AGAGTCTTGCCTGTATCCTC
GAPDH	GGAAGGTGAAGGTCGGAGT	TGGGTGGAATCATATTGGAA

## Supplementary Materials

The following are available online. Figure S1: HPLC-DAD of standards phenylethanoids glycosides used in this study; Table S1: Main representative chemical structures of phenylethanoid glycosides of *V. thapsus* leaf aqueous extract.

## Sample Availability

Samples of the compounds are not available from the authors.
