# Supplementary material for: Phytochemical Analysis and Anti-Inflammatory and Anti-Osteoarthritic Bioactive Potential of Verbascum thapsus L. (Scrophulariaceae) Leaf Extract Evaluated in Two In Vitro Models of Inflammation and Osteoarthritis
Source: Molecules. 2021 Sep 5;26(17):5392. doi: 10.3390/molecules26175392 (PMC8434610; doi:10.3390/molecules26175392)
Supplement: Supplementary file 1 [file molecules-26-05392-s001.zip › molecules-1338587-supplementary.pdf]

Table S1. Main representative chemical structures of phenylethanoid glycosides of *V. thapsus* leaf aqueous extract.

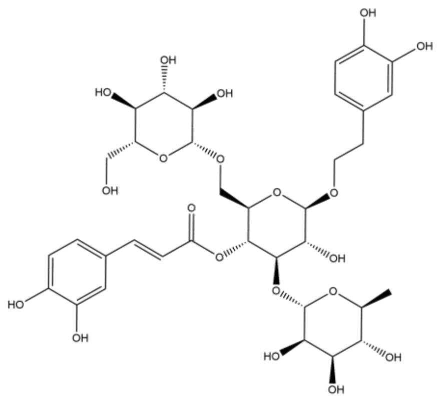

(1) samioside

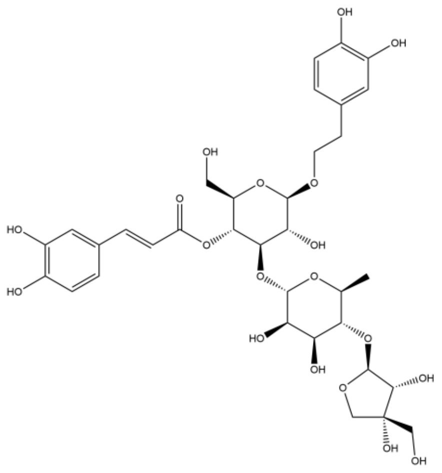

(2) echinacoside

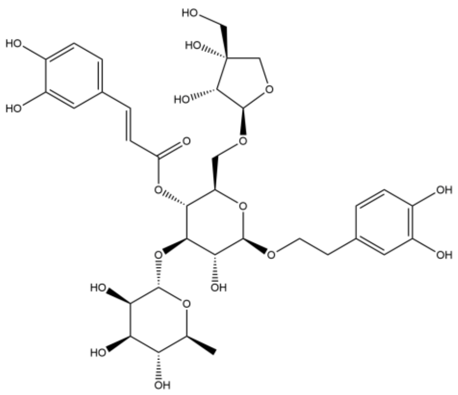

(3) forsythoside B

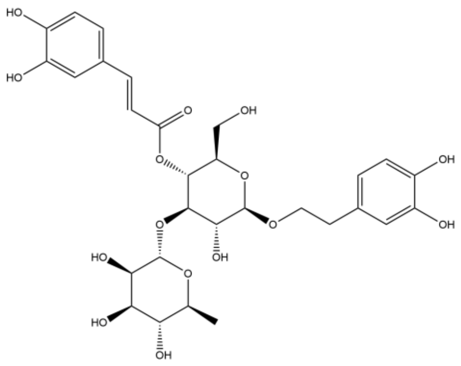

(4) verbascoside

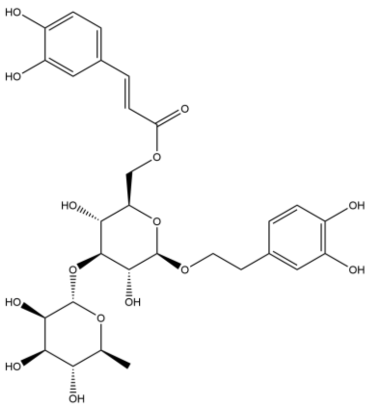

(5) iso-verbascoside

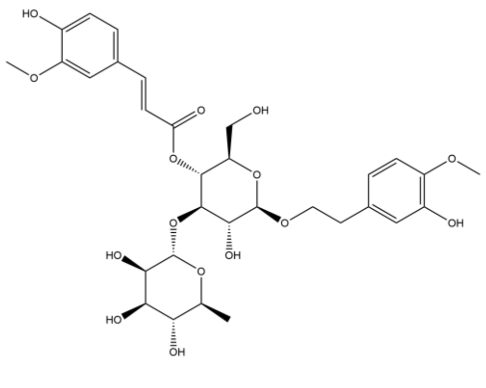

(6) martynoside

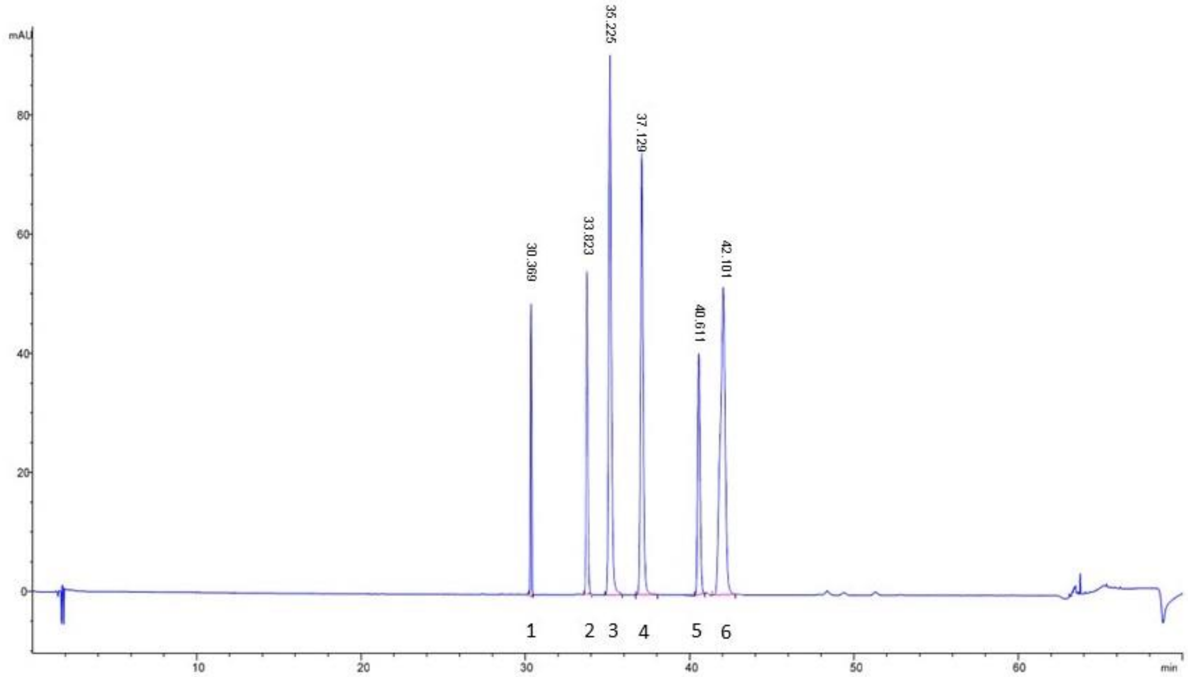

Figure S1. HPLC-DAD of standards phenylethanoids glycosides [**1** samaoside (rt 30.369, purity 80% by HPLC); **2** echinacoside (rt 33.823, purity 98% by HPLC); **3** forsythoside B (rt 35.225, purity 95% by HPLC); 4 verbascoside (rt 37.129, purity 91% by HPLC); **5** isoverbascoside (rt 40.611, purity 84% by HPLC); **6** martynoside (rt 42.101, purity 98% by HPLC)] used to identify and quantify the phenylethanoids fingerprint of *V. thapsus* leaf aqueous extract. Column: Ascentis Express C18, 15 cm×4.6 mm, 2.7 µm d.p. The numbers indicating peaks refer to the identified and quantified compounds reported in Table 1.

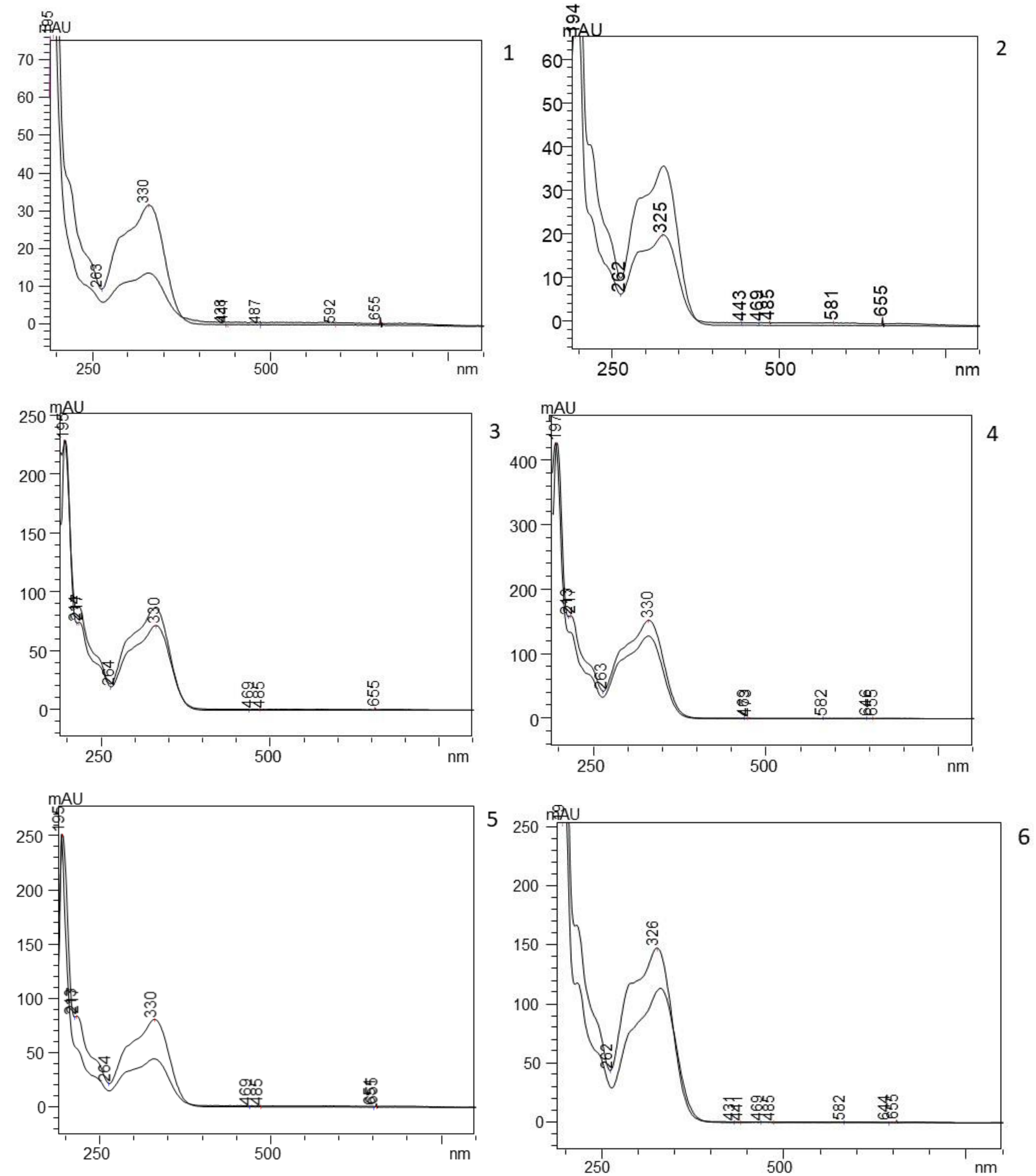

DAD UV spectral data of standards and phenylethanoids analytes identified in *V. thapsus* leaf aqueous extract.
